# Comparison of leaf anatomy and essential oils from *Drimys brasiliensis* Miers in a montane cloud forest in Itamonte, MG, Brazil

**DOI:** 10.1186/s40529-014-0041-y

**Published:** 2014-05-10

**Authors:** Bruna Paula da Cruz, Evaristo Mauro de Castro, Maria das Graças Cardoso, Katiúscia Freire de Souza, Samísia Maria Fernandes Machado, Patrícia Vieira Pompeu, Marco Aurélio Leite Fontes

**Affiliations:** 1 Departamento de Biologia, Universidade Federal de Lavras, Campus Universitário, Lavras, CEP 37200-000 MG Brazil; 2 Departamento de Química, Universidade Federal de Lavras, Campus Universitário, Lavras, CEP 37200-000 MG Brazil; 3 Departamento de Química, Universidade Federal de Sergipe, Avenida Marechal Rondon, s/n, São Cristóvão, CEP 49100-000 SE Brazil; 4 Departamento de Ciências Florestais, Universidade Federal de Lavras, Campus Universitário, Lavras, CEP 37200-000 MG Brazil

**Keywords:** Drimys brasiliensis, Altitude, Cloud Forest, Essential oils, Leaf anatomy

## Abstract

**Background:**

*Drimys brasiliensis* Miers is native to Brazil, where it is mainly found in montane forests and flooded areas in the South and Southeast regions of the country. The objectives of the present study were to compare the leaf anatomy and the chemical constitution of the essential oils from *D. brasiliensis* present in two altitude levels (1900 and 2100 m), in a Montane Cloud Forest, in Itamonte, MG, Brazil.

**Results:**

A higher number of sclereids was observed in the mesophyll of the leaves at 1900 m altitude. At 2100 m, the formation of papillae was observed on the abaxial surface of the leaves, as well as an increase in the stomatal density and index, a reduction in leaf tissue thickness, an increase in the abundance of intercellular spaces in the mesophyll and an increase in stomatal conductance and in carbon accumulation in the leaves. Fifty-nine constituents have been identified in the oils, with the predominance of sesquiterpenes. Two trends could be inferred for the species in relation to its secondary metabolism and the altitude. The biosyntheses of sesquiterpene alcohols at 1900 m, and phenylpropanoids and epi-cyclocolorenone at 2100 m, were favored.

**Conclusions:**

*D. brasiliensis* presented a high phenotypic plasticity at the altitude levels studied. In relation to its leaf anatomy, the species showed adaptive characteristics, which can maximize the absorption of CO_2_ at 2100 m altitude, where a reduction in the partial pressure of this atmospheric gas occurs. Its essential oils presented promising compounds for the future evaluation of biological potentialities.

**Electronic supplementary material:**

The online version of this article (doi:10.1186/s40529-014-0041-y) contains supplementary material, which is available to authorized users.

## Background

The Mantiqueira Mountains are a part of the Atlantic Forest domain in Brazil, in which altitudinal forests are located, and can be classified as Cloud Forests, since they are practically covered by fog during most of the year. These forests represent biodiverse and endemic unique ecosystems, which are generally fragmented and threatened, and present a vegetation which is not well studied yet (Aldrich et al. [1997]; Bertoncello et al. [2011]). Studies conducted by Bertoncello et al. ([2011]) showed that *Drimys brasiliensis* Miers is considered one of the indicator species of cloud forests in the South and Southeast regions of Brazil. In addition, studies by Meireles et al. ([2008]) suggested that the species presents its best development in montane environments.

*D. brasiliensis* is an aromatic species, which belongs to the Winteraceae family. It is native to Brazil, where it is popularly known as *cataia* or *casca-d’anta* and it is mainly found in montane forests and flooded areas in the South and Southeast regions of the country (Lorenzi and Matos [2008]; Souza and Lorenzi [2008]). The species has a peculiar anatomic structure among the angiosperms of the Brazilian flora, as it does not present vessel elements on the xylem, and the stomatal pores are clogged with plugs, comprised of cutin and wax (Feild et al. [2000]; Souza and Lorenzi [2008]; Marquínez et al. [2009]).

The essential oils from *D. brasiliensis* are rich in sesquiterpenes, which call the attention because of the variety of biological activities that they present, such as anti-bacterial, anti-inflammatory, anti-allergic and anti-fungal (Limberger et al. [2007]; Ribeiro et al. [2008]; Lago et al. [2010]). The production of essential oils in plants and other secondary metabolites is constantly influenced by environmental factors (Gobbo-Neto and Lopes [2007]), which also cause modifications in the leaf anatomic structures of the plants (Kofidis et al. [2007]; Zarinkamar et al. [2011]). In altitudinal forests, atmospheric pressure, temperature, radiation, humidity and wind speed are some of the factors that suffer alterations with the increase in the altitudinal gradient and directly influence vegetation (Körner [2007]).

Evaluations of leaf anatomy, such as analyses of stomatal density and index, are essential for the comprehension of the gas exchange between the plants and the atmosphere. They become relevant mainly if the new scenarios of climatic changes for the future are considered (Zhou et al. [2012]), with the increase in the atmospheric CO_2_ concentration, among other impacts (Joly [2007]). The objectives of the present study were to compare the leaf anatomy and the chemical constitution of the essential oils from *D. brasiliensis* present in two altitude levels (1900 and 2100 m), in a Montane Cloud Forest in the Mantiqueira Mountains, in Itamonte, MG, Brazil.

## Methods

### Plant material

Leaves and branches from *Drimys brasiliensis* Miers were collected in a Montane Cloud Forest, above the 1500 m altitude level (Veloso et al. [1991]), inside the Mantiqueira Mountains, Atlantic Forest domain, in the city of Itamonte, South of the state of Minas Gerais, Brazil. The collection was carried out in January 2013 at two altitude levels (1900 and 2100 m). The leaves and branches were collected in adult plants, in the east face of the crown. Voucher species were deposited at the ESAL Herbarium of Universidade Federal de Lavras under numbers 27307 and 27216.

The study area is situated at a Private Natural Heritage Reserve, called RPPN Alto-Montana, and integrates the Mantiqueira Mountains Environmental Preservation Area, characterized by a Federal Conservation Unit for Sustainable Use. The approximate geographic coordinates of the area are 22°21′55″S e 44°48′32″W. The area is situated 15 km away from the entrance of the National Park of Itatiaia [*Parque Nacional do Itatiaia*].

The climate in the city of Itamonte according to the Köppen classification is Cwb - mesothermal climate with dry winter, mild summer and rainy season in the summer. The average temperature of the warmest month is less than 17.3°C and the lowest is more than 12.7°C (Sá Júnior et al. [2012]). The historical average annual rainfall is 1749 mm. The predominant soil is cambisol (Pane and Pereira [2005]).

### Characterization of leaf anatomy

The anatomic characterization was carried out in Laboratório de Anatomia Vegetal of Departamento de Biologia from Universidade Federal de Lavras, Brazil. Fully expanded leaves were selected, free of pathogens, from 4 individuals of *D. brasiliensis*/altitude level (1900 and 2100 m). Paradermal and cross sections were carried out on 3 leaves/individual and 5 fields/leaf were analyzed.

The paradermal sections were carried out on both leaf surfaces (adaxial and abaxial), manually, with the help of a steel blade. The sections were clarified in a 50% sodium hypochlorite solution for about 1 minute and then they were washed in distilled water two times for 10 minutes, and flushed with 1% safranin. The sections were mounted on slides and coverslips with 50% glycerin (Kraus and Arduin [1997]) and photographed in an Olympus CX 31 optical microscope, coupled to a digital camera. The stomatal analysis was carried out on the abaxial surface of the leaves, where the stomata were present, through the image analysis software UTHSCSA-Imagetool*®*, version 3.0. Density (number of stomata per mm^2^), functionality (stomatal polar diameter/stomatal equatorial diameter) and stomatal index [(number of stomata/number of stomata + number of epidermal cells) 100] were measured according to Pereira et al. ([2011]).

The cross sections were carried out from approximately 2 cm^2^ fragments, taken from the region that contains the central vein. In order to obtain permanent slides, leaf fragments were dehydrated in increasing series of ethanol and preserved in 70% ethylic alcohol. The inclusion was made in hydroxyethyl methacrylate Leica*®*, according to the manufacturer’s modified protocol. The samples were sectioned in a semi-automatic microtome, the sections were submitted to staining with toluidine blue and the slides were mounted in Permount*®* (Feder and O’Brien [1968]). The sections were photographed in a Zeiss optical microscope, coupled to a digital camera (AxioCam ERc5s) and analyzed through the UTHSCSA-Imagetool*®* software, version 3.0; the characteristics of the leaf tissues were measured.

### Histochemical tests

Histochemical tests were carried out in Laboratório de Anatomia Vegetal of Departamento de Biologia from Universidade Federal de Lavras, Brazil, according to the methodology proposed by Figueiredo et al. ([2007]). The tests were applied on 3 leaves/individual of *D. brasiliensis*, being 4 individuals/altitude level (1900 and 2100 m). The cross sections were carried out from approximately 2 cm^2^ fragments, taken from the region that contains the central vein with the help of an LPC bench microtome.

For the detection of total lipids, the reagent Sudan IV was used; for the detection of the essential oils, the reagent NADI was used, and for the verification of the phenolic compounds, ferric chloride was used. The sections were mounted on slides and coverslips with 50% glycerin, and photographed in an Olympus CX 31 optical microscope, coupled to a digital camera.

### Scanning electron microscopy

In order to observe the surfaces of *D. brasiliensis* leaves, samples obtained at 1900 and 2100 m altitude were fixed in a Karnovsky solution until the analysis. In Laboratório de Microscopia Eletrônica e Análise Ultraestrutural of Departamento de Fitopatologia from Universidade Federal de Lavras, Brazil, the samples were washed in a 0.05 M cacodylate buffer (3 times - 10 minutes each) and post-fixed in 1% osmium tetroxide, during 4 hours, at room temperature. Later, they were dehydrated in increasing series of acetone (25%, 50%, 75%, 90% and 100%, 3 times - 10 minutes each), submitted to the critical point of CO_2_ desiccation, in a BAL-TEC, CPD-030 equipment, and fixed on a metal support with silver adhesive and recovered with metal gold (10 nm) in a BAL-TEC, SCD-050 device. The prepared leaf material was observed and electro-micrographed in a LEO EVO 40 XVP scanning electron microscope.

### Leaf gas exchange measurements

Leaf gas exchange characteristics from 4 individuals of *D. brasiliensis*/altitude level (1900 and 2100 m) were evaluated, with an infrared gas analyzer - IRGA (LI-6400XT, LI-COR, USA), in 4 fully expanded leaves/individual, starting at 10 o’clock. Stomatal conductance (g_s_), leaf transpiration rate (E) and internal carbon rate (Ci) were evaluated in the leaves.

### Extraction of the essential oils

The extractions of the essential oils were carried out in Laboratório de Química Orgânica - Óleos Essenciais of Departamento de Química from Universidade Federal de Lavras, Brazil. The hydrodistillation method was used, with a Clevenger-type apparatus coupled to a glass flask with round bottom and capacity of 1000 mL, according to the methodology proposed by Brazilian Pharmacopeia (Brasil [2010]).

The extractions of the oils were carried out from fresh leaves, fresh branches and dry leaves from 4 individuals of *D. brasiliensis*/altitude level (1900 and 2100 m), considering that the weight of each plant material was previously standardized. The whole plant material was cleaned with tissue paper. The leaves were cut in small and uniform pieces and the branches were cut in pieces of approximately 3 cm. Drying was carried out in a forced air circulation oven (Fanem Model 320 - SE), at temperatures between 30 and 34°C.

The leaves and the branches were placed in the glass flask and covered with water. The flasks were heated (100 ± 5°C) with the help of heating blankets. The extraction process was carried out in a period of 2 hours; the solution was kept boiling. The oil was then separated from the hydrolate by centrifugation, using a bench centrifuge with an horizontal cross arm (Fanem Baby®I Model 206 BL) at 965,36 × G for 5 minutes. The oil was withdrawn with the help of a Pasteur pipette, placed in a dark glass flask wrapped with aluminum foil and stored under refrigeration.

In parallel with the extractions, a humidity test was carried out, according to Pimentel et al. ([2008]), for a further calculation of the extraction yield. Five grams of each plant material (fresh leaves and fresh branches) were used, submerged in 50 mL cyclohexane in a round bottom glass flask with capacity 250 mL, which was coupled to a condenser with a Dean Stark apparatus (distillation trap). The flask was heated (100 ± 5°C) with the help of a heating blanket. After 2 hours, the water volume present in the plant material was quantified. The humidity was calculated considering the water content in 100 g sample.

Each extraction yield was calculated and expressed in oil weight per plant material weight with Humidity Free Base (% p/p HFB) (Guimarães et al. [2008]).

### Quantitative and qualitative analyses of the essential oils

The quantitative and qualitative analyses of the essential oils were carried out in Departamento de Química from Universidade Federal de Sergipe, Brazil. Quantitative analyses were carried out in a Shimadzu GC-17A gas chromatograph equipped with a flame ionization detector (GC-FID), under the following operational conditions: ZB-5MS fused silica capillary column (5% dimethylpolysiloxane), with 30 m × 0.25 mm i.d. × 0.25 μm film, using helium as a carrier gas, with 1.2 mL min^−1^ flow rate. The temperature was kept at 50°C for 2 min, followed by an increase of 4°C min^−1^, until achieving 200°C. Then, it was increased by 15°C min^−1^, until achieving 300°C, and this temperature was kept for 15 min. The injector temperature was 250°C and the detector temperature (or interface), was 280°C. The injected sample volume was 0.5 μL in ethyl acetate.

Qualitative analyses were carried out in a Shimadzu QP 5050A gas chromatograph coupled to a mass spectrometer (GC-MS), equipped with a J&W Scientific fused silica capillary column (5%-phenyl-95%-dimethylpolysiloxane), with 30 m × 0.25 mm i.d. × 0.25 μm film, using helium as a carrier gas, with 1.2 mL min^−1^ flow rate. The chromatographic conditions of the analyses were the same as the ones used for GC-FID. The MS operational conditions were: ionic capture detector operating by electronic impact and impact energy at 70 eV; scanning speed 1000; scanning interval 0.50 fragments per second and fragments detected in the range of 40 to 500 Da.

The identification of the constituents was carried out based on the comparison of its retention indexes with the ones in the literature (Adams [2007]). For the calculation of the retention index, the Dool and Kratz equation ([1963]) was used in relation to homologous series of n-alkanes (nC_9_-nC_18_). Two equipment libraries have also been used (NIST107 and NIST21), which allow the comparison of the obtained spectrum data with those existing in the libraries.

### Microclimatic data

The microclimatic data were provided by collaborators from Departamento de Ciências Florestais from Universidade Federal de Lavras, Brazil, and refer to the period of one year follow-up by a meteorological station of the brand WatchDog model 2900ET, installed in each altitude level (1900 and 2100 m).

### Statistical analyses

The data related to *D. brasiliensis* leaf anatomy and leaf gas exchanges were submitted to a variance analysis, and the means were compared by the Scott-Knott test at 95% confidence, using the statistical program SISVAR, version 4.6 (Ferreira [2003]). For the leaf anatomy analysis, the results obtained from 5 field/leaf were considered, being 3 leaves/individual of *D. brasiliensis* and 4 individuals/altitude level (1900 and 2100 m) and, for the leaf gas exchange analysis, the data obtained from 4 leaves/individual were considered, being 4 individuals/altitude level.

Due to the big quantity of chemical compounds present in the essential oils from *D. brasiliensis*, the PCA technique (Principal Component Analysis) was chosen to check the similarity between the essential oils from the fresh leaves (FF), dry leaves (FS) and fresh branches (G) in two altitude levels, regarding the proportions of their chemical constituents. The analysis was carried out using the program CHEMOFACE (Nunes et al. [2012]). For this analysis, the means of the proportions of the chemical constituents of the oils from 4 individuals of *D. brasiliensis*/altitude level (1900 and 2100 m) were calculated.

## Results and discussion

### Morpho-anatomical properties of *D. brasiliensis* leaves

**Figure 1:**
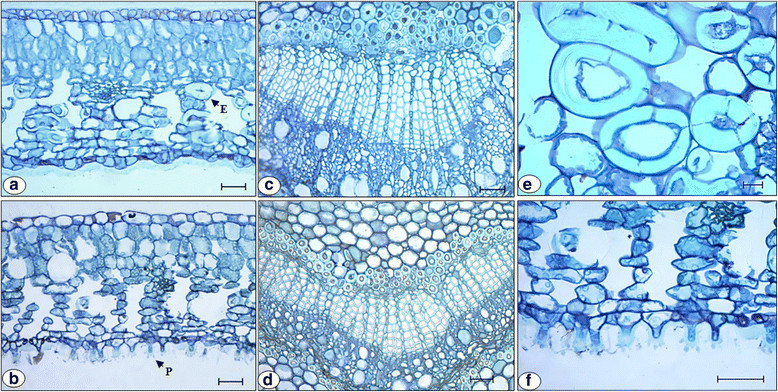
**Cross sections of** ***Drimys brasiliensis*** **Miers leaves collected at 1900 m (a, c, e) and 2100 m (b, d, f) altitude in a Montane Cloud Forest in Itamonte, MG, Brazil, evidencing the central vein of the leaves (c, d), the sclereids (e) and the papillose epidermis (f).** E = A sclereid, P = A papilla. Bars: 50 μm **(a, b, c, d, f)**, 20 μm **(e)**.

In the cross section, *D. brasiliensis* leaves presented a uniseriate epidermis, externally coated by a cuticle, whose thickness ranged according to the altitude, and a dorsiventral mesophyll, with a palisade parenchyma in which there were three cell layers, and a spongy parenchyma with six to eight layers (Figure 1a, b). In both altitude levels, the stomata found were of the paracitic type and were present only on the abaxial surface of *D. brasiliensis* leaves; these were classified as hypostomatal, which is in accordance with the descriptions for the representatives of the Winteraceae family (Feild et al. [1998]; Feild et al. [2000]).

In the mesophyll, sclerenchyma cells of the sclereid type were found with round and elliptical shape (Figure 1a, e). Metcalfe and Chalk ([1957]) report the presence of such sclereids in the representatives of the Winteraceae family. Sclereids were present in a higher amount in *D. brasiliensis* leaves collected at 1900 m altitude (Table 1), while intercellular spaces occurred in higher abundance in the mesophyll of the leaves at 2100 m (Figure 1b; Table 1). The central vein of *D. brasiliensis* leaves collected at 1900 m altitude (Figure 1c), presented many sclereids like the ones in the mesophyll, in addition to a higher number of secretory cavities (Table 1).

**Table 1:** **Anatomical characteristics of** ***Drimys brasiliensis*** **Miers leaf tissues present at 1900 and 2100 m altitude in a Montane Cloud Forest in Itamonte, MG, Brazil**

	1900	2100
LL (μm)	433.55 ± 53.05a	330.94 ± 32.34b
CT (μm)	12.43 ± 3.34a	7.98 ± 1.04b
PP (μm)	130.51 ± 21.24a	102.64 ± 9.23b
SP (μm)	200.02 ± 21.09a	146.44 ± 20.96b
SCL	10.60 ± 0.96a	3.35 ± 0.44b
AIS	0.11 ± 0.01a	0.22 ± 0.01b
SC	7.88 ± 0.47a	4.20 ± 0.69b

LL, Thickness of the leaf lamina; CT, Thickness of the cuticle of the adaxial surface; PP, Thickness of the palisade parenchyma; SP, Thickness of the spongy parenchyma; SCL, Number of sclereids in the mesophyll; AIS, Abundance of intercellular spaces in the mesophyll (Area/Area); SC, Number of secretory cavities near the central vein. All values are expressed as the mean ± standard deviation. Means followed by the same letter in rows (a, b) do not differ by the Scott-Knott test (P < 0.05).

The histochemical tests carried out in this study helped identify cavities and cells or secretory idioblasts with oil content in *D. brasiliensis* leaves. These structures were found in both altitude levels. The presence of secretory cells has already been reported for the species of the Winteraceae family (Metcalfe and Chalk [1957]; West [1969]; Esau [1974]; Read and Menary [2000]). The histochemical tests also identified phenolic compounds in the mesophyll of *D. brasiliensis* leaves.

**Figure 2:**
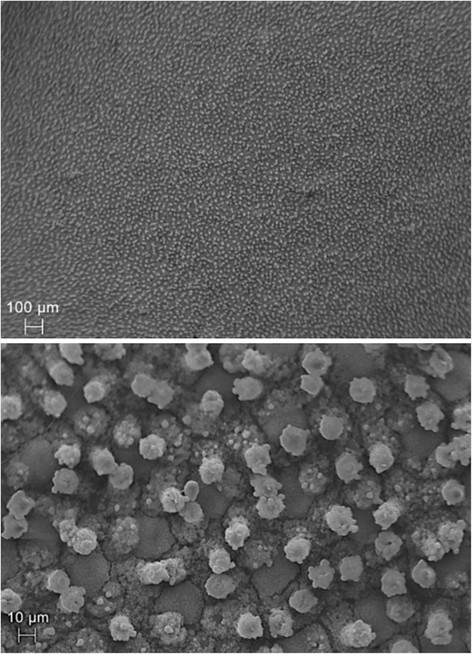
**Scanning electron microscopy of the abaxial surface of** ***Drimys brasiliensis*** **Miers leaves collected at 2100 m altitude in a Montane Cloud Forest in Itamonte, MG, Brazil, evidencing the papillose epidermis.**

The abaxial surface of *D. brasiliensis* leaves collected at 2100 m altitude presented abundant papillae with variable length (Figure 1b, f; Figure 2). These structures were not found in the leaves collected at 1900 m. Smith ([1943]), Metcalfe and Chalk ([1957]), Ehrendorfer et al. ([1979]), Feild et al. ([1998]), Feild et al. ([2000]) and Eller et al. ([2013]) reported the presence of papillae on the abaxial surface of *Drimys* sp*.* leaves; however, the authors did not present any differences in the occurrence of these epidermal structures in relation to biotic and abiotic factors. Vieira and Gomes ([1995]) attributed the function to converge light stimuli for the mesophyll to the papillae of the abaxial epidermis from *Psychotria leiocarpa* Cham. and Schltdl., *P. stenocalyx* Müll. Arg. and *P. tenuinervis* Müll. Arg. (Rubiaceae) leaves*,* for the performance of photosynthesis, since the studied leaves developed under reduced luminosity.

Recent studies conducted by Eller et al. ([2013]) proved that *D. brasiliensis* is able to absorb water from the fog through the cuticle of the leaves and, according to these authors, this is an essential process for the wide distribution of the species in cloud forests. The authors suggested that the water absorption in *D. brasiliensis* leaves occurs mainly on the adaxial surface, and the abaxial surface is responsible for gas exchange during and after the presence of the fogs, because of its hydrophobic characteristics and because it contains most of the stomata.

Feild et al. ([1998]), studying the species *Drimys winteri* var. *chilensis* (DC) A. Gray, evidenced that the plugs of the stomata (constituted of cuticle material) present on the abaxial surface of *Drimys* leaves, are responsible for the protection of the leaves against excessive soaking caused by the recurring fog in cloud forests. The authors discuss that the plugs, together with the papillae and epicuticular waxes, due to their high hydrophobicity, comprise a structure which is able to prevent the constant formation of water films on the leaves, that would make gas exchange difficult. The authors also clarify that the diffusion of CO_2_ in water is much slower than in the air (about 10000 times), so a water film on the leaves would reduce gas exchange, because it would create a barrier for the entrance of CO_2_ in the leaves. Brewer and Smith ([1997]), evaluating 50 montane species at 2400 m altitude in the USA, also discussed this issue, attributing a fundamental role regarding the avoidance of water film formation on the leaves to the leaf trichomes.

Based on the presented evidences, it can be inferred that the occurrence of papillae in *D. brasiliensis* individuals at 2100 m altitude is possibly related to the higher humidity at this altitude level (Table 2) and to the reduction of the partial pressure of CO_2_ with the increase in altitude (Körner [2007]). The papillae present on the abaxial surface of the leaves would favor the absorption of CO_2_ through the same process described for *D. winteri* by Feild et al. ([1998]), i.e. comprising a hydrophobic matrix, which would avoid the formation of a water film on the leaves. Complementing these data, the leaf transpiration of *D. brasiliensis* was higher at 2100 m altitude (Table 3), preventing the accumulation of water on the leaves and compensating the higher humidity at this altitude level (Table 2). Our results confirm the proposal of Feild et al. ([1998]) and aggregate knowledge, and also present the phenotypic plasticity of *D. brasiliensis* in altitudinal forests, being able to alter/favor the formation of papillae as a compensatory mechanism for the reduction of the partial pressure of CO_2_ at higher altitudes.

**Table 2:** **Average microclimate parameters in two altitude levels (1900 and 2100 m) in a Montane Cloud Forest in Itamonte, MG, Brazil**

	1900	2100
Radiation (watt/m^2^)	11.2	24.6
Sun hours (h)	8.0	8.6
Air relative humidity (%)	81.6	83.5
Maximum humidity (%)	91.3	94.0
Minimum humidity (%)	68.0	67.0
Temperature (°C)	13.1	11.8
Maximum temperature (°C)	16.6	16.0
Minimum temperature (°C)	10.1	8.4
Maximum extreme temperature (°C)	24.1	23.8
Minimum extreme temperature (°C)	0.3	−1.7
Wind speed (km/h)	0.4	0.5
Maximum wind speed (km/h)	11.0	11.0
Dew point (°C)	9.7	8.7
Maximum dew point (°C)	17.6	17.3
Minimum dew point (°C)	−18.1	−21.3

Follow-up carried out during a period of one year by meteorological stations (WatchDog model 2900ET) installed in each altitude level.

**Table 3:** **Gas exchanges of** ***Drimys brasiliensis*** **Miers leaves present at 1900 and 2100 m altitude in a Montane Cloud Forest in Itamonte, MG, Brazil**

	1900	2100
g_s_ (mmol_CO2_.m^−2^.s^−1^)	0.20 a	0.46 b
Ci (g/mol)	187.62 a	394.54 b
E (mmol_H2O_.m^−2^.s^−1^)	3.14 a	7.18 b

g_s_, Stomatal conductance; Ci, Internal carbon; E, Transpiration rate. Means followed by the same letter in rows (a, b) do not differ by the Scott-Knott test (P < 0.05).

Other results, which complement this hypothesis, are presented in Tables 3 and 4. Through the analysis of these results, it is possible to observe that the average stomatal density and index of *D. brasiliensis* individuals at 2100 m altitude were statistically higher than the ones at 1900 m (Table 4). The strategy of the species would be to invest in a higher stomatal production to compensate the lower availability of atmospheric CO_2_ at 2100 m altitude. Furthermore, the stomatal conductance at 2100 m altitude presented values that were superior to those at 1900 m, facilitating the accumulation of carbon in the leaves (Table 3). This accumulation was also facilitated by the greater abundance of intercellular spaces in the mesophyll of the leaves at 2100 m (Table 1). Thus, it can be proposed that the hydrophobic surface formed by the papillae (Figure 1b, f; Figure 2) could act together with the increase in the number of stomata (Table 4) and the higher stomatal conductance (Table 3), establishing an adaptive strategy, which would favor gas exchange in *D. brasiliensis* leaves, compensating the reduction in the partial pressure of CO_2_ at 2100 m altitude.

**Table 4:** **Stomatal analysis of** ***Drimys brasiliensis*** **Miers present at 1900 and 2100 m altitude in a Montane Cloud Forest in Itamonte, MG, Brazil**

	1900	2100
SE (μm)	19.09 ± 0.81a	18.89 ± 0.74a
SP (μm)	26.67 ± 1.48a	26.49 ± 2.20a
SD (stomata/mm^2^)	222.02 ± 22.11a	305.86 ± 46.69b
SI (%)	14.56 ± 0.99a	18.91 ± 2.70b
SF (SP/SE)	1.40 ± 0.07a	1.40 ± 0.10a

SE, Stomatal equatorial diameter; SP, Stomatal polar diameter; SD, Stomatal density; SI, Stomatal index; SF, Stomatal functionality. All values are expressed as the mean ± standard deviation. Means followed by the same letter in rows (a, b) do not differ by the Scott-Knott test (P < 0.05).

The differentiation of stomata is genetically determined and suffers the influence of environmental factors, such as the concentration of atmospheric CO_2_, temperature, humidity and luminosity (Li et al. [2006]; Zhao et al. [2008]; Zhou et al. [2012]). Different authors report the positive correlation between the increase in the number of stomata and the elevation in altitude for several species, such as *Eucalyptus pauciflora* Sieb. ex Spreng. (Körner and Cochrane [1985]), *Vaccinium myrtillus* L. (Woodward [1986]), *Clinopodium vulgare* L. (Kofidis et al. [2007]) and *Nepeta nuda* L. (Kofidis and Bosabalidis [2008]).

According to Kouwenberg et al. ([2007]), the reduction in the partial pressure of CO_2_ plays an essential role in the increase in stomatal density for many species with the elevation in altitude. The authors discuss that, among climatic factors, which suffer alterations with altitude, such as atmospheric pressure, temperature, humidity, radiation and wind speed, only the atmospheric pressure is altered in a way that does not depend on the microclimate conditions of each environment.

Through the analysis of the results described in Table 1, it can be noted that, at 2100 m altitude, the average thickness of all *D. brasiliensis* leaf tissues were statistically lower than the ones at 1900 m. It can be suggested that this reduction in thickness could favor the diffusion of CO_2_ in the leaves by the reduction in the routes to be run by this gas until the chloroplasts. This process would be even more facilitated by the increase in the abundance of intercellular spaces in the mesophyll of the leaves at 2100 m (Table 1), reducing the resistance in the intercellular spaces.

According to Taiz and Zeiger ([2009]), the main entrance routes for CO_2_ in the leaves during photosynthesis are the stomata and, after crossing this gap, CO_2_ needs to diffuse through the mesophyll until it finds the chloroplasts. The authors explain that each point on this route imposes resistance to the diffusion of CO_2_ in the leaves, and the main ones are: resistance of the borderline layer, stomatal resistance, resistance in intercellular spaces and resistance of the liquid phase.

Studies conducted by Nautiyal and Purohit ([1980]) with *Artemisia sp.* at two altitudes (550 and 3600 m) evidenced that the elevation in altitude contributed to the reduction in leaf thickness. According to the authors, this reduction in leaf thickness would negatively affect the ability of the plants to avoid the beginning of hydric stress at higher altitudes; however, it could increase CO_2_ capture.

Analyzing cuticle thickness separately, *D. brasiliensis* leaves collected at 1900 m altitude presented a cuticle with approximately 12.43 μm, and the ones collected at 2100 m, with 7.98 μm (Table 1). The increase in cuticle thickness is a common adaptive response to species that grow in environments with high levels of radiation, such as the ones that survive at higher altitudes (Shepherd and Griffiths [2006]; Jacobs et al. [2007]). Due to this fact, considering the increase in radiation at 2100 m altitude isolately (Table 2), it was expected that, at this altitude level, *D. brasiliensis* leaves would present a higher cuticle thickness on the adaxial surface.

These results can be explained by a peculiar characteristic of cloud forests: the presence of constant fog at the vegetation level (Aldrich et al. [1997]). It is known that in cloud forests of the Mantiqueira Mountains, Brazil, the fog occurs for about 65 to 90% of the days (Eller et al. [2013]). At the level of 2100 m altitude, the environment presented a higher relative humidity than at 1900 m (Table 2) and the fog was possibly the most responsible factor for this increase, due to the frequency of its occurrence in cloud forests. Thus, despite the higher radiation levels at 2100 m altitude, the fog could act as a barrier against the direct penetration of solar beams in *D. brasiliensis* leaves at this altitude level, which would minimize the actual radiation falling upon the leaves and, in a way, it would reduce the need of protection performed by the cuticle.

Aldrich et al. ([1997]) explain that, in tropical montane cloud forests, the fog influences the vegetation because it reduces the incidence of solar radiation, causes the soaking of tree crowns and suppresses evapotranspiration. Future studies will be necessary to prove the real radiation rates that fall upon *D. brasiliensis* leaves, seeking a better comprehension of this interaction mechanism of the species with cloud forests.

### Chemical characterization of the essential oils from *D. brasiliensis*

#### Yield of the essential oils

The yield of the essential oils from *D. brasiliensis* ranged from 0.03 to 1.02% (Table 5). Regardless drying, the leaves presented higher oil yields than the branches.

**Table 5:** **Yield of the essential oils from** ***Drimys brasiliensis*** **Miers present at 1900 and 2100 m altitude in a Montane Cloud Forest in Itamonte, MG, Brazil**

	1900			2100		
Plant material	FF	FS	G	FF	FS	G
Mass (g)	55.77	36.24	80.05	55.77	35.16	80.03
Humidity (%)	67.00	0.00	50.50	53.00	0.00	51.50
^*^Yield (% p/p HFB)	0.92	1.02	0.03	0.48	0.80	0.03

FF, Fresh leaves; FS, Dry leaves; G, Fresh branches; HFB, Humidity free base. ^*^Yield = Yield average of the oils from 4 individuals of *D. brasiliensis*/altitude level.

Ribeiro et al. ([2008]) evidenced a yield of approximately 0.97% for the essential oils from the leaves and stem barks from *D. brasiliensis*. Previously, Limberger et al. ([2007]), studying the essential oils from the species, noted that the yields ranged from 1.4 to 1.5% for the oils from fresh leaves, 1.0% for the oils from dry leaves, 0.4 to 0.6% for the oils from stem barks and 0.4% for the oils from unripe fruits. Gomes et al. ([2013]) found, for the same species, a yield of approximately 0.3% for the oil from fresh leaves.

No studies were found in the literature comparing the essential oils from *D. brasiliensis* in different altitudes. In this study, a trend for a higher oil yield was observed at 1900 m altitude (Table 5). According to Gobbo-Neto and Lopes ([2007]), the production of secondary metabolites in the plants can be influenced by different factors, such as seasonal conditions, temperature, radiation, hydric availability and atmospheric composition. It is known that many of these factors directly influence the development of the plants along altitudinal gradients (Körner [2007]).

Vokou et al. ([1993]), while studying variations in the essential oils from *Origanum vulgare* subsp. *hirtum* Ietsw. in Greece showed that the highest yields of the oils were found for plants that grow at low altitudes. Studies conducted by Kizil ([2010]) evidenced similar results for *Thymbra spicata* var. *spicata* L. in some regions in Turkey. These results were attributed to the higher temperatures in the regions with a lower altitude.

According to Gobbo-Neto and Lopes ([2007]), the production of essential oils seems to be favored by higher temperatures, although too high temperatures lead to an excessive loss of these metabolites. Thus, based on the literature, it is possible to suggest that the production of essential oils from *D. brasiliensis* may have been favored by the higher temperatures at 1900 m altitude (Table 2), where the species presented a trend to a higher yield of the essential oils from fresh and dry leaves (Table 5). New studies with the species at controlled microclimate conditions may support this hypothesis.

#### Chemical composition of the essential oils

**Figure 3:**
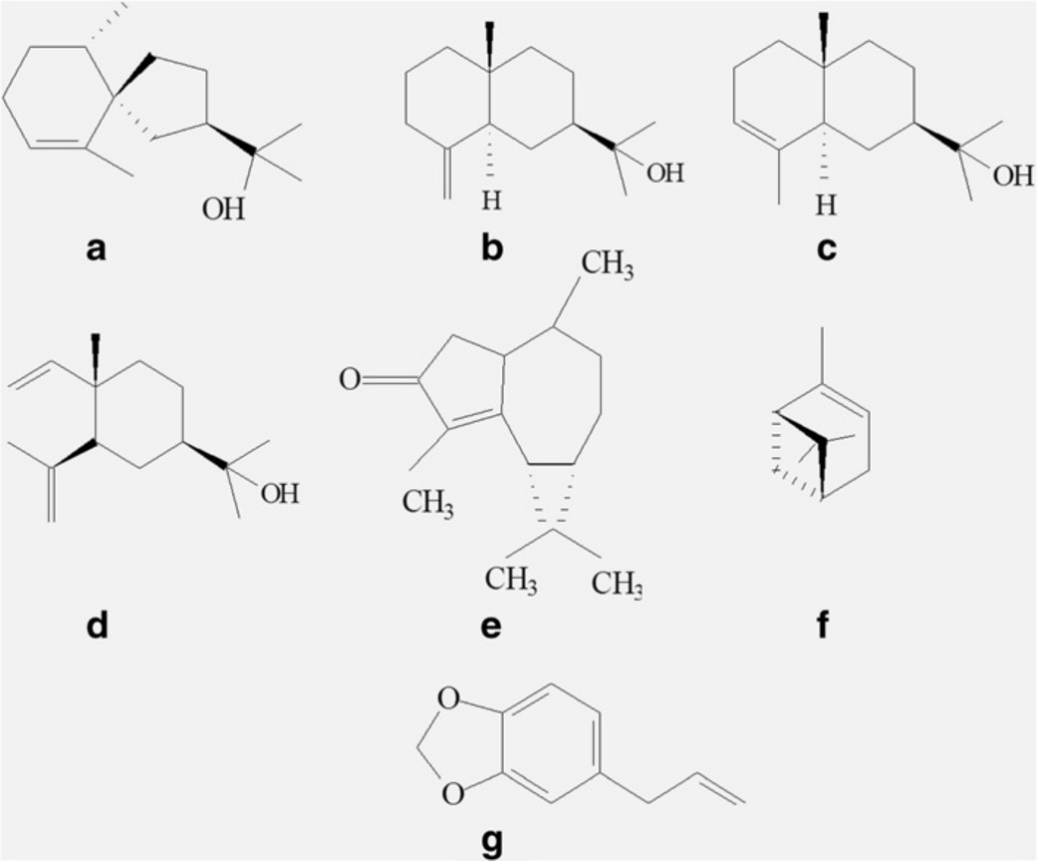
**Chemical structures of the major constituents in the essential oils from** ***Drimys brasiliensis*** **Miers. (a)** Hinesol; **(b)** β-Eudesmol; **(c)** α-Eudesmol; **(d)** Elemol; **(e)** Epi-Cyclocolorenone; **(f)** α-Pinene; **(g)** Safrole.

Fifty-nine chemical compounds have been identified, representing from 92.81 to 98.75% of the constitution of the essential oils from *D. brasiliensis* (Table 6). The major compounds were hinesol, β-eudesmol, α-eudesmol, elemol, epi-cyclocolorenone, α-pinene and safrole (Figure 3; Table 6). There was the predominance of sesquiterpenes in all oils, and the highest values were found for the oils from fresh branches at 1900 m altitude (81.46%). The drimanic sesquiterpenes drimenol and polygodial, common for *Drimys* oils (Cicció [1997]; Malheiros et al. [2001]; Muñoz-Concha et al. [2007]), were not detected.

**Table 6:** **Chemical composition of** ***Drimys brasiliensis*** **Miers essential oils present at 1900 and 2100 m altitude in a Montane Cloud Forest in Itamonte, MG, Brazil**

	Compound	RT	TKI	CKI	FF 1900 (%)	FF 2100 (%)	FS 1900 (%)	FS 2100 (%)	G 1900 (%)	G 2100 (%)
	Monoterpene Hydrocarbons				13.63	9.73	21.53	19.03	11.76	7.30
1	α-Thujone	6.499	924	939	0.17	0.03	0.29	0.15	0.03	-
2	α-Pinene	6.745	932	945	8.39	6.12	13.27	11.54	9.84	6.20
3	Camphene	7.186	946	955	-	0.02	0.08	0.04	-	-
4	Sabinene	7.926	969	973	0.53	0.55	0.86	1.11	0.28	0.17
5	β-Pinene	8.070	974	977	1.96	1.50	3.02	2.73	1.53	0.93
6	Myrcene	8.425	988	985	0.47	0.25	0.76	0.62	-	-
7	α-Terpinene	9.402	1014	1010	0.32	0.15	0.53	0.42	-	-
8	ρ-Cymene	9.701	1020	1019	0.33	0.11	0.17	0.25	-	-
9	o-Cymene	9.705	1022	1019	-	-	0.36	-	-	-
10	Limonene	9.866	1024	1023	0.56	0.39	0.25	-	0.08	-
11	Sylvestrene	9.869	1025	1023	-	-	0.44	1.03	-	-
12	γ-Terpinene	11.029	1054	1056	0.73	0.44	1.16	0.66	-	-
13	Terpinolene	12.252	1086	1091	0.17	0.17	0.34	0.48	-	-
	**Oxygenated Monoterpenes**				**2.33**	**1.29**	**2.45**	**1.91**	**5.53**	**3.19**
14	1,8-Cineole	9.990	1026	1027	-	-	0.08	0.15	-	-
15	Linalool	12.715	1095	1103	-	-	-	-	0.07	-
16	Camphor	14.754	1146	1149	-	-	0.06	-	0.08	0.04
17	Terpinen-4-ol	16.220	1174	1182	1.92	1.00	2.16	1.38	3.64	1.29
18	p-Cimen-8-ol	16.591	1179	1190	-	-	-	-	-	0.21
19	α-Terpineol	16.827	1186	1201	0.41	0.29	0.15	0.38	1.74	1.65
	**Sesquiterpene Hydrocarbons**				**2.99**	**7.48**	**7.27**	**14.48**	**0.07**	**0.25**
20	β-Elemene	25.869	1389	1396	0.21	0.20	0.19	0.46	-	-
21	α-Funebrene	26.415	1402	1409	-	-	0.15	0.15	-	-
22	α-Gurjunene	26.702	1409	1415	-	0.54	-	1.94	-	-
23	α-Cedrene	26.857	1410	1419	-	-	-	0.11	-	-
24	(E)-Caryophyllene	27.135	1417	1425	-	-	1.11	0.55	-	-
25	β-Caryophyllene	27.137	1417	1425	0.60	0.40	-	-	-	-
26	α-trans-Bergamotene	27.736	1432	1439	-	-	-	0.09	-	-
27	Aromadendrene	27.979	1439	1445	-	-	0.13	-	-	-
28	cis-Muurola-3,5-diene	28.452	1448	1456	-	-	-	0.08	-	-
29	α-Humulene	28.617	1452	1460	-	-	0.39	0.18	-	-
30	(E)-β-Farnesene	28.630	1454	1460	-	0.48	-	0.37	-	-
31	(E)-9-epi-Caryophyllene	28.940	1464	1467	-	-	-	0.13	-	-
32	trans-Cadina-1(6),4-diene	29.441	1475	1479	-	-	-	0.16	-	-
33	γ-Curcumene	29.644	1481	1483	0.35	2.86	2.36	5.32	-	0.12
34	β-Selinene	30.044	1489	1493	-	-	0.30	0.09	-	-
35	trans-Muurola-4(14),5-diene	30.311	1493	1499	-	-	-	0.17	-	-
36	Bicyclogermacrene	30.484	1500	1503	1.69	2.03	2.30	2.73	0.07	0.13
37	β-Curcumene	30.995	1514	1515	-	0.55	0.26	0.68	-	-
38	δ-Cadinene	31.543	1522	1529	0.14	0.42	0.08	1.14	-	-
39	Zonarene	31.664	1528	1532	-	-	-	0.13	-	-
	**Oxygenated Sesquiterpenes**				**74.62**	**59.92**	**56.56**	**50.23**	**81.39**	**73.90**
40	Elemol	32.749	1548	1558	6.72	6.08	4.62	4.60	9.91	4.01
41	(E)-Nerolidol	33.127	1561	1568	-	0.15	0.10	-	-	0.09
42	Palustrol	33.487	1567	1577	-	0.22	-	0.35	-	0.03
43	Spathulenol	33.916	1577	1587	0.70	1.06	0.68	0.86	1.65	3.23
44	Viridiflorol	34.509	1592	1602	-	1.02	-	0.53	0.25	0.18
45	Ledol	34.942	1602	1613	-	-	-	0.16	-	-
46	10-epi-γ-Eudesmol	35.615	1622	1630	0.45	0.29	0.10	0.19	0.09	-
47	β-Acorenol	36.023	1636	1640	-	-	-	-	-	0.91
48	Hinesol	36.290	1640	1647	21.42	15.20	15.85	11.91	20.61	8.73
49	α-Cadinol	36.954	1652	1664	-	-	-	0.20	-	-
50	β-Eudesmol	37.016	1649	1665	26.16	15.92	19.63	12.70	29.27	11.10
51	α-Eudesmol	37.112	1652	1668	19.17	13.53	15.37	10.78	19.61	8.69
52	Epi-β-Bisabolol	37.456	1670	1676	-	0.92	0.21	0.63	-	0.31
53	β-Bisabolol	37.459	1674	1677	-	-	-	-	-	0.15
54	Epi-Cyclocolorenone	41.114	1774	1775	-	5.53	-	7.32	-	36.47
	**Phenylpropanoids**				**2.02**	**14.39**	**7.11**	**7.48**	**0.00**	**8.95**
55	Safrole	21.334	1285	1295	1.87	11.84	5.42	7.48	-	5.04
56	Eugenol	24.435	1356	1364	-	-	0.34	-	-	0.12
57	Methyl Eugenol	26.439	1403	1409	0.15	-	-	-	-	-
58	Myristicin	31.658	1517	1532	-	2.55	1.35	-	-	2.94
59	2,6-Dimethoxy-4-allylphenol	34.956	^*^	1613	-	-	-	-	-	0.85
	**Total identified (%)**				**95.59**	**92.81**	**94.92**	**93.13**	**98.75**	**93.59**

RT, retention time in minutes provided by GC-MS; TKI, tabulated Kovats index (Adams, [2007]); CKI, calculated Kovats index; FF, fresh leaf oils; FS, dry leaf oils; G, fresh branch oils; %, percentage of the component. ^*^Source: WILEY8.

Lago et al. (2010) evidenced the predominance of sesquiterpenes in the oils from D. brasiliensis leaves (52.31%) collected in Campos do Jordão, SP, Brazil, and the major constituents were α-cedrene (6.87%), bicyclogermacrene (5.31%), τ-muurolol (7.75%) and drimenol (9.96%). In the oils from the stem barks, the authors showed that monoterpenes were present in higher proportions (90.02%). Ribeiro et al. (2008) also observed the predominance of sesquiterpenes (66.0%) in the oils from fresh leaves and stem barks from *D. brasiliensis* collected in Rio Grande do Sul, Brazil. Studies conducted by Limberger et al. (2007) showed that in the oils from fresh and dry leaves of *D. brasiliensis*, equivalent contents of monoterpenes (31.2% to 53.9%) and sesquiterpenes (37.1% to 65.4%) were found and, in the oils from fruits and stem barks, sesquiterpenes were predominant (75.5% to 93.4%).

In addition to sesquiterpenes, monoterpenes and phenylpropanoids were also found in the essential oils from leaves and branches of *D. brasiliensis* (Table 6). The formation of sesquiterpenes and monoterpenes occurred by the mevalonic acid and the 1-deoxy-D-xylulose-5-phosphate (DXPS) biosynthetic pathways, while the formation of phenylpropanoids occurred by the shikimic acid pathway (Solomons and Fryhle [2002]). The highest proportions of monoterpenes were found in the oils from dry leaves (23.98% at 1900 m and 20.94% at 2100 m altitude) (Table 6). Phenylpropanoids seem to be the constituents that most responded to the elevation in altitude. In the fresh leaves and in the fresh branches, this response was even more evident, because at 1900 m, phenylpropanoids were identified in the proportions of 2.02% and 0%, respectively. At 2100 m, the proportions increased to 14.39% and 8.95% (Table 6). It is thus inferred that the elevation in altitude favored the biosynthesis of these compounds in *D. brasiliensis.*

A great part of phenylpropanoids is derived from the cinnamic acid, which is formed from the phenylalanine amino acid by the deamination activity of phenylalanine ammonia-lyase (PAL) (Dixon and Paiva [1995]; Dixon et al. [2002]; Ziaei et al. [2012]). Phenylalanine ammonia-lyase plays a fundamental role in the regulation of the production of phenylpropanoids in plants. It is known that many factors, such as plant age, herbivores, UV radiation and low temperatures, affect its biosynthesis and, consequently, the synthesis of phenylpropanoids (Ziaei et al., [2012]).

Many phenylpropanoids are produced in response to any biotic or abiotic stress. The biosynthesis of some of these compounds is stimulated in injured plants and plants under the attack of herbivores. Others, such as anthocyanins and flavonoids, increase their concentrations in response to higher rates of visible and UV radiation. The biosynthesis of many of them is favored by low temperatures and nutritional stress; however, the influence of these factors on the production of phenylpropanoids is not certain yet (Dixon and Paiva [1995]).

It is important to point out that, most of the times, the production of secondary metabolites in plants is influenced by different factors acting together (Gobbo-Neto and Lopes [2007]). Thus, it is proposed that the temperature is possibly one of the factors that might influence the chemical composition of the essential oils from *D. brasiliensis*. The lower temperatures at 2100 m altitude (Table 2), might have probably favored the route of the phenylpropanoids.

In this study, the phenylpropanoids identified were safrole, eugenol, methyl eugenol, myristicin and 2,6-dimethoxy-4-allylphenol (Table 6). Except for 2,6-dimethoxy-4-allylphenol, all these compounds have already been found in the essential oils from *D. brasiliensis* (Limberger et al. [2007]; Ribeiro et al. [2008]; Lago et al. [2010]; Gomes et al. [2013]).

#### Principal Component Analysis (PCA)

**Figure 4:**
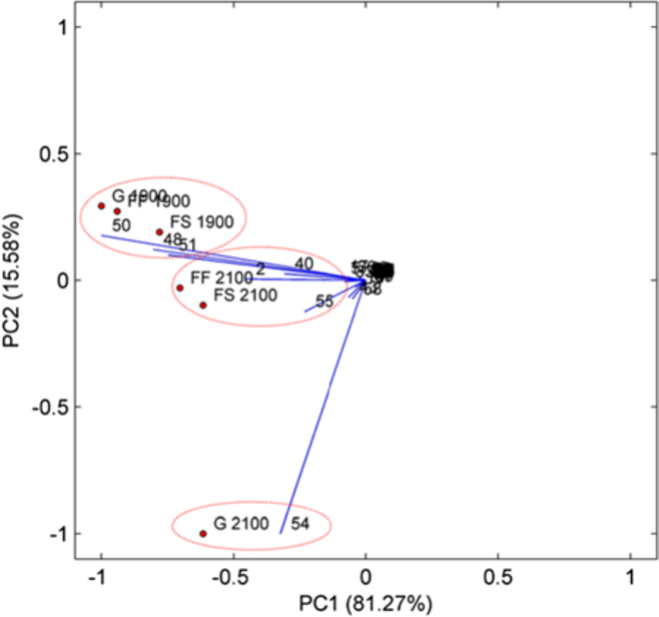
**Biplot graph PC1 x PC2 of the loadings and the scores for the essential oils from fresh leaves (FF), dry leaves (FS) and fresh branches (G) of** ***Drimys brasiliensis*** Miers present at 1900 and 2100 m altitude in a Montane Cloud Forest in Itamonte, MG, Brazil, in relation to the proportions of their chemical constituents.^*^The constituents correspond to the numbers presented in Table 6.

The analysis of the essential oils through the PCA technique showed that, with the first principal component and the second principal component, it was possible to describe 96.85% of the data, considering 81.27% of total variance described by the first principal component (Figure 4). PCA allows separating the volatile oils from *D. brasiliensis* in three groups, which express the similarities and the differences of these oils in relation to their chemical constituents.

Observing Figure 4, it can be seen that the oils from the fresh branches at 2100 m altitude are different from the other groups by the compound 54, epi-cyclocolorenone, a sesquiterpene ketone (Figure 3e). Epi-cyclocolorenone was biosynthesized only at 2100 m altitude in all oils, and in higher proportions in the oils from fresh branches (36.47%), followed by dry leaves (7.32%) and fresh leaves (5.53%) (Table 6). Thus, it can be seen that the elevation in altitude favored the biosynthesis of this compound in *D. brasiliensis*, in addition to favoring the formation of phenylpropanoids*.* It can also be inferred that epi-cyclocolorenone, possibly, presents biosynthesis in the branches and is translocated to the leaves.

Studies by Limberger et al. ([2007]), Ribeiro et al. ([2008]) and Gomes et al. ([2013]) evidence the presence of cyclocolorenone as a major constituent of the oils from *D. brasiliensis*. Epi-cyclocolorenone is different from cyclocolorenone only by the presence of a 1,1-dimethylcyclopropane group oriented backwards. Cyclocolorenone occurs rarely in nature and it has already been reported in *Pseudowintera colorata* (Raoul) Dandy and *Tasmannia* sp., belonging to the Winteraceae family, in addition to *Solidago canadensis* L. (Asteraceae), *Ledum palustre* L. (Ericaceae) and *Magnolia grandiflora* L. (Magnoliaceae) (Ribeiro et al. [2008]).

The oils from the fresh and dry leaves at 2100 m altitude were separated by the presence of the compounds 2, 40 and 55 (α-pinene, elemol and safrole) (Figure 3f, d, g; Figure 4). It can be highlighted that safrole was biosynthesized in the leaves in higher proportions at 2100 m altitude and, in the branches, it was formed only at 2100 m (Table 6), evidencing that the elevation in altitude favored the production of phenylpropanoids in *D. brasiliensis.*

The other group was formed by all oils from *D. brasiliensis* at 1900 m altitude, separated by the compounds 48, 50 and 51, hinesol, β-eudesmol and α-eudesmol, respectively (Figure 3a, b, c; Figure 4). These constituents are classified as tertiary sesquiterpene alcohols, characterized by the presence of one hydroxyl group connected to one tertiary carbon (Solomons and Fryhle [2002]) and they were biosynthesized in higher proportions at 1900 m altitude in all essential oils (Table 6). These results confirm the predominance of the sesquiterpenes in the oils from the species.

The presence of these sesquiterpene alcohols among the major constituents of the oils from *D. brasiliensis* becomes relevant due to the diversity of biological potentialities already described for these compounds. Miyazawa et al. ([1996]) demonstrated the antimutagenic potential of β-eudesmol extracted from *Dioscorea japonica* Thunb. (Dioscoreaceae) rhizomes. Recent studies by Jalali et al. ([2013]), mentioned that α-eudesmol demonstrated to be a compound able to induce apoptosis in tumor cells. In addition, Ouyang et al. ([2012]) mentioned that hinesol proved to be a compound that inhibits gastric secretion in rats. This compound also proved able to improve the circulation and metabolism of the brain.

## Conclusions

*D. brasiliensis* presented a high phenotypic plasticity at the altitude levels studied. In relation to its leaf anatomy, the species showed adaptive characteristics, which can maximize the absorption of CO_2_ at 2100 m altitude, where a reduction in the partial pressure of this atmospheric gas occurs. These characteristics were the formation of papillae on the abaxial surface of the leaves, an increase in the stomatal density and index, a reduction in leaf tissue thickness, an increase in the abundance of intercellular spaces in the mesophyll and an increase in stomatal conductance and in carbon accumulation in the leaves.

The essential oils from *D. brasiliensis* presented yields between 0.03 and 1.02%, and the highest values were found for the leaves. Fifty-nine chemical constituents were identified in the oils, with the predominance of sesquiterpenes. Two trends could be inferred for the species in relation to its secondary metabolism and the altitude. The biosyntheses of sesquiterpene alcohols at 1900 m, and phenylpropanoids and epi-cyclocolorenone at 2100 m, were favored.

This research stimulates new studies that investigate the adaptive characteristics of *D. brasiliensis* in altitudinal forests. In addition, its essential oils presented promising compounds for the future evaluation of biological potentialities.

## Authors’ original submitted files for images

Below are the links to the authors’ original submitted files for images. Authors’ original file for figure 1 Authors’ original file for figure 2 Authors’ original file for figure 3 Authors’ original file for figure 4
