# Supplementary figures and images for: Comparison of leaf anatomy and essential oils from Drimys brasiliensis Miers in a montane cloud forest in Itamonte, MG, Brazil
Source: Bot Stud. 2014 May 10;55:41. doi: 10.1186/s40529-014-0041-y (PMC5432844; doi:10.1186/s40529-014-0041-y)

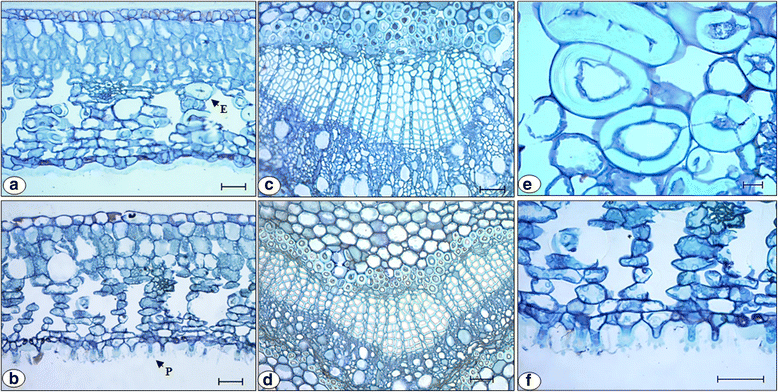

Supplement: Supplementary file 1 — Authors’ original file for figure 1 [file 40529_2014_41_MOESM1_ESM.gif]

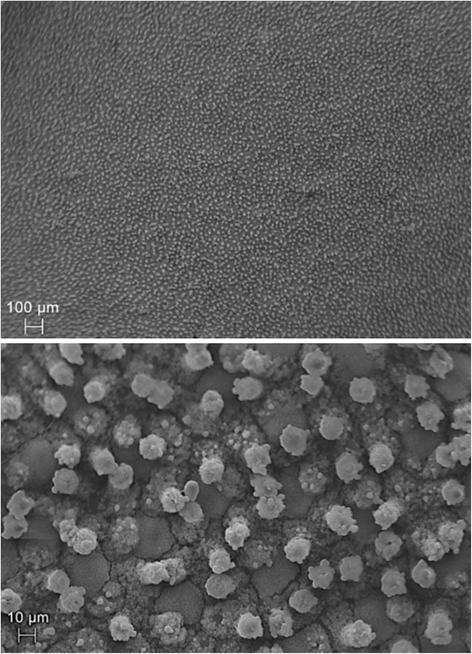

Supplement: Supplementary file 2 — Authors’ original file for figure 2 [file 40529_2014_41_MOESM2_ESM.gif]

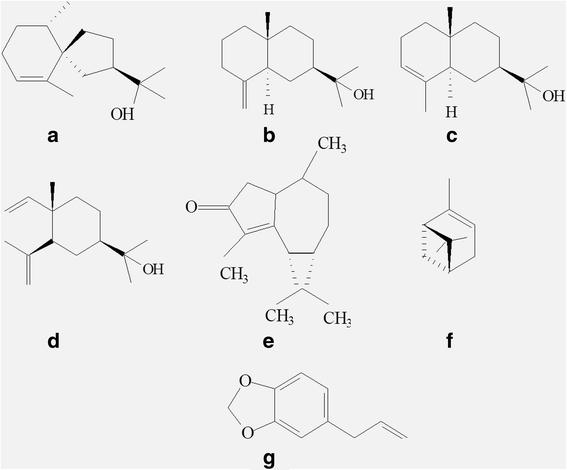

Supplement: Supplementary file 3 — Authors’ original file for figure 3 [file 40529_2014_41_MOESM3_ESM.gif]

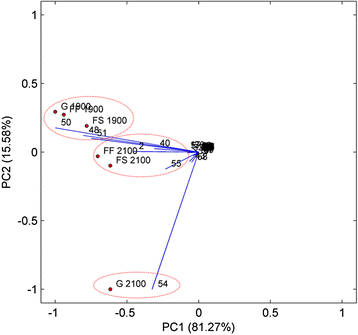

Supplement: Supplementary file 4 — Authors’ original file for figure 4 [file 40529_2014_41_MOESM4_ESM.gif]
